# *Diatraea saccharalis* history of colonization in the Americas. The case for human-mediated dispersal

**DOI:** 10.1371/journal.pone.0220031

**Published:** 2019-07-24

**Authors:** Fabricio J. B. Francischini, Erick M. G. Cordeiro, Jaqueline B. de Campos, Alessandro Alves-Pereira, João Paulo Gomes Viana, Xing Wu, Wei Wei, Patrick Brown, Andrea Joyce, Gabriela Murua, Sofia Fogliata, Steven J. Clough, Maria I. Zucchi

**Affiliations:** 1 Graduate Program in Genetics and Molecular Biology, Institute of Biology, University of Campinas, Campinas, Brazil; 2 Department of Entomology and Acarology, University of Sao Paulo, Luiz de Queiroz College of Agriculture (USP/ESALQ), Piracicaba, São Paulo, Brazil; 3 Department of Plant Biology, Institute of Biology, University of Campinas, Campinas, Brazil; 4 Department of Crop Science, University of Illinois, Urbana, Illinois, United States of America; 5 Department of Public Health, University of California, Merced, California, United States of America; 6 Instituto de Tecnología Agroindustrial del Noroeste Argentino, Estación Experimental Agroindustrial Obispo Colombres, Consejo Nacional de Investigaciones Científicas y Técnicas (ITANOA-EEAOC-CONICET), Tucumán, Argentina; 7 US Department of Agriculture-Agricultural Research Service, Urbana, Illinois, United States of America; 8 Laboratory of Conservation Genetics and Genomics, Agribusiness Technological Development of São Paulo (APTA), Piracicaba, São Paulo, Brazil; United States Department of Agriculture, UNITED STATES

## Abstract

The sugarcane borer moth, *Diatraea saccharalis*, is one of the most important pests of sugarcane and maize crops in the Western Hemisphere. The pest is widespread throughout South and Central America, the Caribbean region and the southern United States. One of the most intriguing features of *D*. *saccharalis* population dynamics is the high rate of range expansion reported in recent years. To shed light on the history of colonization of *D*. *saccharalis*, we investigated the genetic structure and diversity in American populations using single nucleotide polymorphism (SNPs) markers throughout the genome and sequences of the mitochondrial gene cytochrome oxidase (COI). Our primary goal was to propose possible dispersal routes from the putative center of origin that can explain the spatial pattern of genetic diversity. Our findings showed a clear correspondence between genetic structure and the geographical distributions of this pest insect on the American continents. The clustering analyses indicated three distinct groups: one composed of Brazilian populations, a second group composed of populations from El Salvador, Mexico, Texas and Louisiana and a third group composed of the Florida population. The predicted time of divergence predates the agriculture expansion period, but the pattern of distribution of haplotype diversity suggests that human-mediated movement was most likely the factor responsible for the widespread distribution in the Americas. The study of the early history of *D*. *saccharalis* promotes a better understanding of range expansion, the history of invasion, and demographic patterns of pest populations in the Americas.

## Introduction

The ability to move from deteriorated environments to more resourceful ones is a fundamental component of the insects’ life history and has a significant impact on agricultural ecosystems [1]. As colonizing insects arrive at a new location, both neutral (i.e., bottleneck and founder effect) and adaptive variations (i.e., natural selection) will cause genetic divergence to occur between the newly founded population and the ancestral one. In the case of natural selection, selection pressures imposed by host plants and by the environment promote adaptive evolution upon the insects’ morphological, physiological, and behavioral attributes, resulting in divergence of settlers. [2,3]. In other words, the alleles favored by natural selection gradually replace those which are unfavored, changing populations from within [4–6]. The study of genome-wide single nucleotide polymorphisms (SNPs) can be useful to detect traces of selection sweeps and population divergence, and thus be used to address important ecological and evolutionary issues [4–6]. For instance, the careful investigation of the patterns of genetic diversity can contribute to studies on the early demographic history of the pest insects, including past movement and adaptive changes. The mechanism of insect dispersion can be divided into two broad categories: (1) active movement (i.e., migratory and dispersal behavior) [7] and passive movement [8]. Passive movement can be further divided into human-mediated [9–11] and non-human mediated movement—passively transported by boats, trucks or trains in the former, or by wind or water in the latter [12,13]. In both cases of passive movement, the direction of the displacement is biased and correlates with features of the natural environment or human activities.

Substantial advancement has been made in recent years to understand patterns of insect movement [14]. Such studies can be particularly useful in the context of pest insects [15–18], where the information about the origin of infestations and the patterns of dispersal have a direct impact on pest management strategies, including insecticide resistance management. *Diatraea saccharalis* (Fabricius) (Lepidoptera: Crambidae) is one of the most critical sugarcane and maize pests in the Western Hemisphere [19]. This insect is responsible for considerable crop damage and economic losses and is widely distributed throughout South and Central America, the Caribbean region, and the southern United States [20–23]. Although studies have addressed the distribution of this pest, the underlying details of the history of colonization in the Americas remain mostly unknown. The putative center of origin of *D*. *saccharalis* seems to be floating grasses (*Paspalum* spp. and *Panicum* spp.) along the delta of the Orinoco river in Venezuela [19]. However, while this is plausible, this hypothesis is still mostly uncorroborated.

An intriguing aspect of the life history of *D*. *saccharalis* is its low dispersal capacity and site fidelity that is suggestive of a more sedentary lifestyle in which matings are mostly restricted to small groups of related insects. Mark-recapture studies recovered over 45% of adults at around 50 meters from the release point, demonstrating the limited dispersal ability of this moth [24,25]. The dispersal distance was slightly increased to around 800 meters when the moths dispersal was aided by the wind. Recent studies have also investigated population structure and gene flow of *D*. *saccharalis* and detected significant genetic structure in samples from North, Central and South America [21,23,26,27]. However, these studies relied on a limited number of genetic markers (i.e., mitochondrial and SSR markers) and therefore could not directly reveal the dispersion patterns of *D*. *saccharalis* across the Western Hemisphere [26,28]. To summarize the problem in one question: how can an insect with such a limited dispersal ability have occupied large portions of the American continents?

Our primary goal in this study was to investigate the genetic structure and diversity in populations of *D*. *saccharalis* in the Americas using SNPs identified through the genotyping-by-sequencing (GBS) method [29]. This approach can be used to analyze differences in frequencies of neutral and adaptive alleles among populations and to provide a powerful tool to investigate demographic history as well as to identify markers putatively under selection. Such studies give a framework for understanding the evolutionary patterns of the introduced invasive populations of *D*. *saccharalis* in the Americas.

## Material and methods

### Insect collections

A total of 250 specimens of *D*. *saccharalis* were collected in maize and sugarcane production regions in Brazil, Argentina, El Salvador, and the United States during the 2011/2012 and 2012/2013 crop seasons (S1 Table). Upon collection, the individual larvae were kept in Petri dishes containing artificial diet and maintained in controlled condition until pupation (27±1°C; 70% of relative humidity, and a photoperiod of 12D:12L). Healthy pupae were then transferred to cylindrical cages (40 x 30 cm) and kept in solitary conditions at 20±1°C until the adults emerged. Species identification was confirmed by the direct examination of the adults’ genitalia, and only *D*. *saccharalis* were used in this study [30,31]. Moths were then stored at -80°C until further sample processing.

### DNA extraction

The DNA was extracted from the adult moth tissues following the CTAB protocol described by Doyle and Doyle (1990) [32]. The integrity of DNA was evaluated in 0.8% (w/v) agarose gels with 1X TAE buffer (Tris, acetic acid, EDTA, pH 8.0), by concentrations estimated by comparison with known amounts of a DNA standard (λ phage). The gels were stained in an ethidium bromide bath (0.5 mg/mL), and DNA bands were visualized and photographed under UV light.

### Genotyping-by-sequencing

GBS libraries were produced as described by Poland et al. (2012) [33]. DNA concentrations were determined using Picogreen (Molecular Probes, Eugene, Oregon) and a Synergy HT (BioTek, Winooski, Vermont) microplate reader, and adjusted to approximately 50 ng/μl. Five microliters (~250 ng) were pipetted into 96-well plates containing 2.5 μl 0.1 μM specific DNA barcoded *PstI* adaptors. The restriction enzymes *PstI* (New England Biolabs, Ipswich, MA, USA) and *MspI* (New England Biolabs) were used to digest the DNA and to reduce genome complexity. The barcoded *PstI* adapters and a common non-barcoded *MspI* adapter were ligated to the digested DNA and amplified by PCR to create an enriched library. The resulting library was single-end sequenced to 100 bases on a single lane using the Illumina HiSeq 4000 sequencing kit v1 (Illumina, Inc., San Diego, CA, USA). The fastq files obtained were demultiplexed with the bcl2fastq v2.17.1.14 (Illumina) by the Roy J. Carver Biotechnology Center at the University of Illinois at Urbana-Champaign producing nearly 404 million raw reads. The UNEAK pipeline, a multi-sample, SNP-calling approach developed for analyzing GBS data from species without reference genomes [34], was used to analyze the first 64 bases (beginning with the *PstI* restriction site) to identify SNPs.

TASSEL 5 [35] was used to filter SNP data such that each marker was present in at least 70% of the individuals within each population. Using this filtering, we identified 1,331 SNPs for the 250 individuals included in our GBS library.

### Population genomics analyses

File conversion to other population genetics programs was made using PGDSpider v.2.1.0.3 [36]. To identify candidate loci that may have been under selection during the range expansion of *D*. *saccharalis* in the Americas, the LOSITAN program [37], which employs the FDIST2 algorithm [38], was used to detect SNPs that are F_ST_ outliers. This method evaluates the relationship between the expected distribution of F_ST_ and heterozygosity assuming an island model of migration. In LOSITAN analysis, 100,000 simulations were run by using the stepwise mutation model with the option of neutral mean F_ST_. Markers that presented F_ST_ higher than the 95% confidence interval were considered candidates for positive selection, and markers that presented F_ST_ lower than the 95% confidence interval were considered candidates for balancing selection. Genetic diversity and F statistics were estimated under a random model, in which the sampled populations were considered representative of the species and with a common evolutionary history. Allele frequencies, the number of alleles (A), allelic richness (A_R_) the observed heterozygosity (H_O_) and expected heterozygosity (H_E_) and the inbreeding coefficient (F_IS_) were estimated using the R package *diveRsity* [39]. Cluster analysis was based on the construction of dendrograms using Nei’s genetic distance [40], and the neighbor-joining method was performed using the R package *poppr* [41]. The stability of the clusters was tested through 1,000 bootstraps resamples. The genetic structure was investigated employing the non-model-based approach DAPC using the R package *adegenet* [42]. The hierarchical distribution of genomic diversity within and among groups of populations was investigated with Analyses of Molecular Variance (AMOVA) also using the package *poppr*.

### Phylogenetic analysis and divergence time estimation

A total of 167 sequences of a fragment of the cytochrome oxidase subunit I (COI) were retrieved from the BOLD repository (http://www.boldsystems.org/) to offer additional support for the study of the early demographic history of *D*. *saccharalis* in the Americas (S2 Table). DNA sequences were trimmed (533nt) and aligned with MUSCLE in MEGA X [43]. Unique haplotypes were identified in DnaSP software [44], and a phylogenetic analysis based on haplotypes was performed using maximum likelihood (ML) under the HKY+G model was conducted in MEGA X. A total of 1000 bootstraps were used for node support.

Tests for substitution model and molecular clock were also performed in MEGA X (S3 Table). Divergence time estimation was inferred by a Bayesian method implemented in BEAST 1.7.4 [45]. Divergence time calculation has been extensively used for the mitochondrial gene COI; however, the selection of realistic divergence rates is one of the main challenges of such studies. Lack of fossils or a species-specific diverge rate are factors responsible for inconsistencies in the estimation [46]. To overcome those obstacles, we used three different divergence rates frequently used for arthropods. The first rate is the standard rate of 2.3% divergence/My generally used for mitochondria of insects [47]. However, the recent literature suggests examples for slower [48] and faster raters [49,50]. Thus, a second rate of 3.54% [51] and a third rate of 5.2% [52] pairwise divergence per Mya was also used. HKY+G was implemented for the substitution model. The clock model was uncorrelated lognormal and constant size coalescent model for the tree prior. The analysis was run for 50 million generations sampled every 1000 generations in three independent repetitions. A burn-in of 20% was implanted over the total number of trees generated.

## Results and discussion

### GBS sequencing and detection of outlier SNPs

GBS sequencing data returned over 403 million sequence reads, approximately 1.6 million reads per sample. After cleaning and filtering the sequence data, aligning PCR fragments to reveal SNPs, a total of 1331 SNPs were identified to be used in subsequent population genetic analyses for 250 individuals. The F_ST_-outlier analysis showed 125 putative loci under positive selection and 270 under balancing selection (S1 Fig).

The analysis based on outlier SNPs was designed to detect genetic variation putatively driven by natural selection. Populations with shared demographic history should result in similar F_ST_/H_E_ values for all loci, but those that deviate from the null model are therefore candidates for loci under selection [53]. Here, we present evidence that natural selection is acting upon population differentiation; it is not clear, however, which selection pressure prevails, whether it may be environmental, crop management or host related adaptions.

### Genomic diversity

Overall diversity indexes [i.e., number of alleles (A), allelic richness (A_R_), and observed heterozygosity (H_O_)] showed a similar trend; that is, the highest values of genetic diversity were found in Brazil and Argentina whereas the lowest values were found in the U.S. and El Salvador (Table 1). The average values of observed (H_O_) and expected heterozygosity (H_E_) across all populations were 0.12 and 0.16, respectively. The Brazilian population of Jaboticabal-Sugarcane showed the highest observed and expected values (H_O_ = H_E_ = 0.24) (Table 1). The lowest value for the observed and expected heterozygosity was found in the U.S. population of Belle Glade-Sugarcane (H_O_ = 0.03 and H_E_ = 0.08).

**Table 1:** *Diatraea saccharalis* genomic diversity estimates based on 1,331 SNP loci by sampling locations.

Location	N	A	A_R_	H_O_	H_E_	F_IS_
LaCocha_Co	10	1859	1.13	0.1	0.13	0.244
LaCruz_Su	5	1721	1.1	0.1	0.12	0.159
Jujuy_Su	7	1866	1.13	0.11	0.14	0.222
Perga_Co	2	1519	1.05	0.12	0.14	0.183
Qui_Co	4	1552	0.98	0.07	0.13	0.475
Araras_Su	2	1723	1.14	0.16	0.16	0.02
Goias_TBD	4	1856	1.1	0.12	0.17	0.324
Jabo_Su	52	2333	1.41	0.24	0.24	0.006
Morr_Co	4	1910	1.16	0.13	0.17	0.243
MS_TBD	9	2129	1.23	0.14	0.2	0.306
MT_TBD	5	1818	1.12	0.11	0.15	0.225
PAf_Su	3	1751	1.12	0.11	0.15	0.253
Parana_TBD	3	1847	1.16	0.14	0.16	0.175
Pira_Co	25	2118	1.32	0.19	0.19	-0.009
Pira_Su	22	2296	1.38	0.22	0.23	0.046
Rib_Su	4	1916	1.11	0.14	0.18	0.246
Rondo_Co	3	1634	1.03	0.11	0.16	0.347
SHG_Su	4	1916	1.15	0.15	0.19	0.236
SP_TBD	18	2264	1.21	0.13	0.23	0.423
ElNilo_Su	4	1438	1.01	0.05	0.07	0.326
ElPais_Su	2	1277	0.89	0.04	0.13	0.727
BGlade_Su	9	1384	0.95	0.03	0.08	0.629
Louis_Su	15	1526	1.03	0.06	0.09	0.33
Beaum_Su	13	1556	1.07	0.08	0.09	0.149
Wesl_Su	7	1472	1	0.06	0.09	0.361

N = Number of individuals, A = Number of alleles, A_R_ = Allelic Richness, H_O_ = Observed heterozygosity, H_E_ = Expected heterozygosity, F_IS_ = Inbreeding coefficient

The observed values for heterozygosity were less than the expected values in all but the three Brazilian locations (Jaboticabal-Sugarcane, Araras-Sugarcane, and Piracicaba-Maize). Particularly large differences between observed and expected heterozygosity were observed in populations from Argentina and El Salvador (i.e., Quilmes-Maize and El Pais-Sugarcane). Similar values for H_0_ and H_E_ might indicate that most loci are in Hardy-Weinberg equilibrium, which means that the frequency of the alleles tended to remain unchanged over the generations. On the other hand, differences in observed and expected heterozygosity might indicate critical demographic changes.

Overall fewer heterozygotes than expected were found in populations of *D*. *saccharalis*, which was also reflected in the inbreeding coefficient (F_IS_). The average inbreeding coefficient across populations was 0.27, ranging from -0.009 (Piracicaba-Maize) to 0.727 (El Pais-Sugarcane). Departure from the Hardy-Weinberg equilibrium (HWE), as shown previously, and heterozygote deficiency (F_IS_), can be related to different causes. Limited dispersal, well-structured populations, and the evidence for strong positive selection are all factors that point to a more locally confined population dynamic.

### Genetic structure and substructure of *D*. *saccharalis* populations

**Fig 1:**
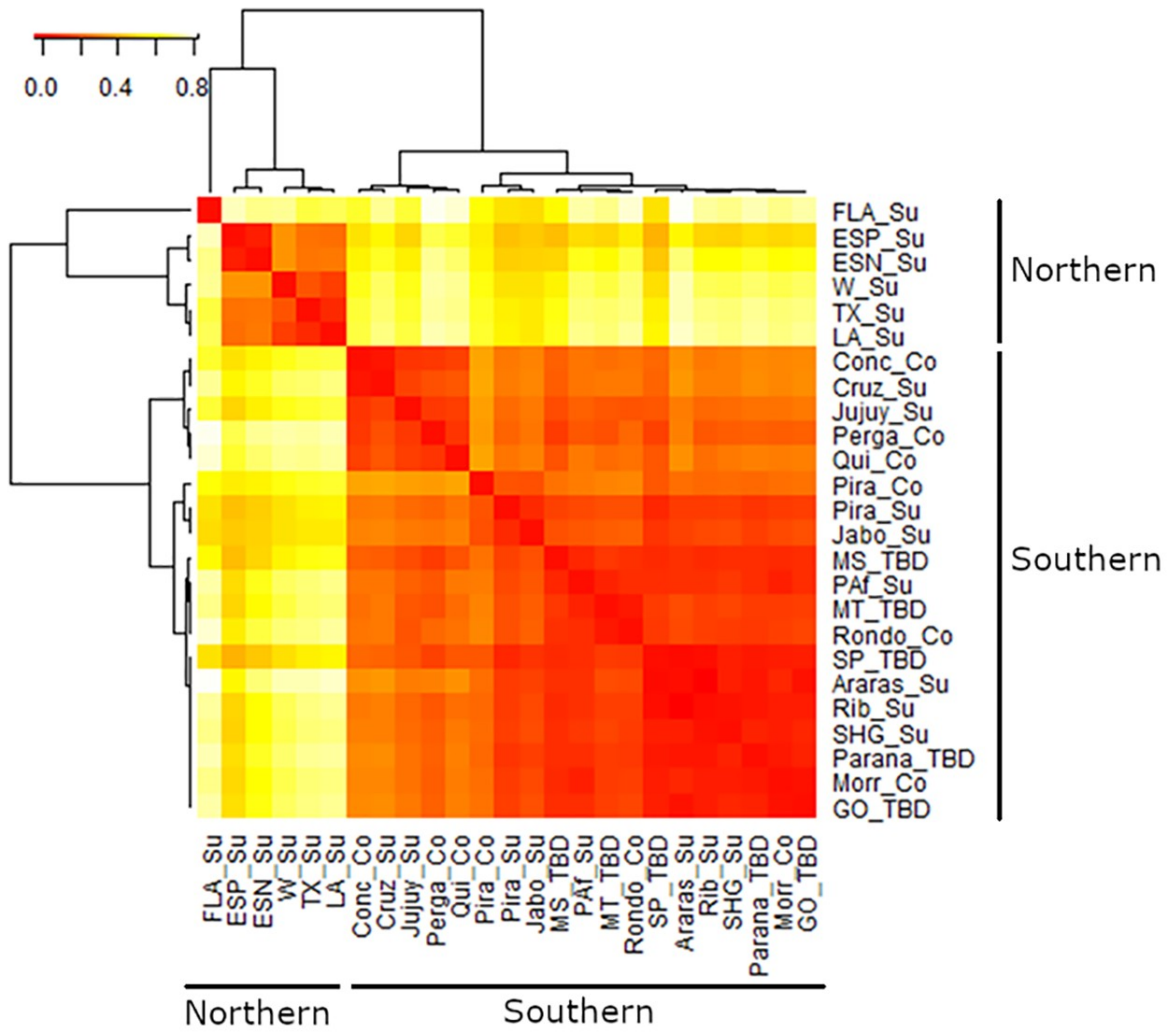
Heatmap of pairwise F_ST_ values between *Diatraea saccharalis* populations, based on 1,331 SNP loci. Dendrograms were plotted using the unweighted-pair-group method with arithmetic mean (UPGMA). Colors indicate the degree of divergence from red (low F_ST_ values, little genetic divergence) to light yellow (high F_ST_ values, strong genetic divergence).

The values of pairwise F_ST_ revealed strong genetic structure between the northern (i.e., U.S. and El Salvador) and the southern (i.e., Brazil and Argentina) parts of the American continents (Fig 1, S4 Table). The highest F_ST_ values were found between the U.S. and the Argentinian populations (Louisiana vs. Buenos Aires, F_ST_ = 0.418). In contrast, an overall low degree of differentiation was found among the Brazilian populations (São Paulo *vs*. Paraná, F_ST_ = 0.003; São Paulo *vs*. Minas Gerais, F_ST_ = 0.004), which can be explained by the short geographical distance and therefore higher gene flow among sampled sites. Alternatively, the differences in genetic structure found in Brazil and the U.S. may be the result of a different pattern of colonization of the two geographical regions. This hypothesis will be developed further in the following paragraphs.

**Fig 2:**
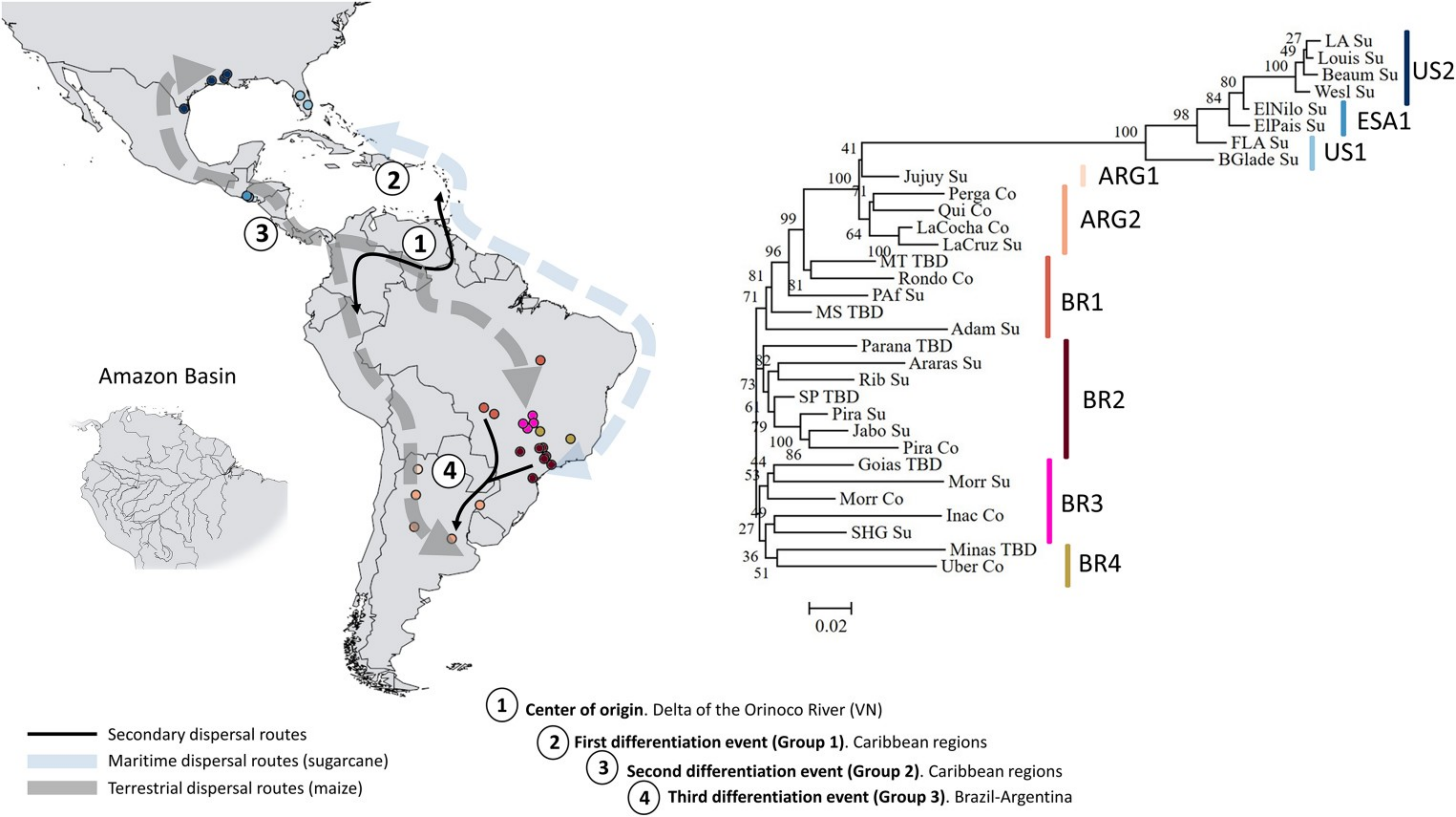
Suggested *D*. *saccharalis* dispersion routes from its center of origin in the Orinoco Basin, Venezuela, based on historical information about maize dispersal and sugarcane introduction, and the genetic relationships observed in this study. The neighbor-joining dendogram showing the relationships among *D*. *saccharalis* populations based on Nei’s genetic distance. A total of 1,331 SNP loci were used, and confidence of nodes was based on 1,000 bootstraps. On the map, numbers indicate major differentiation events and arrows represent putative routes for dispersal.

**Fig 3:**
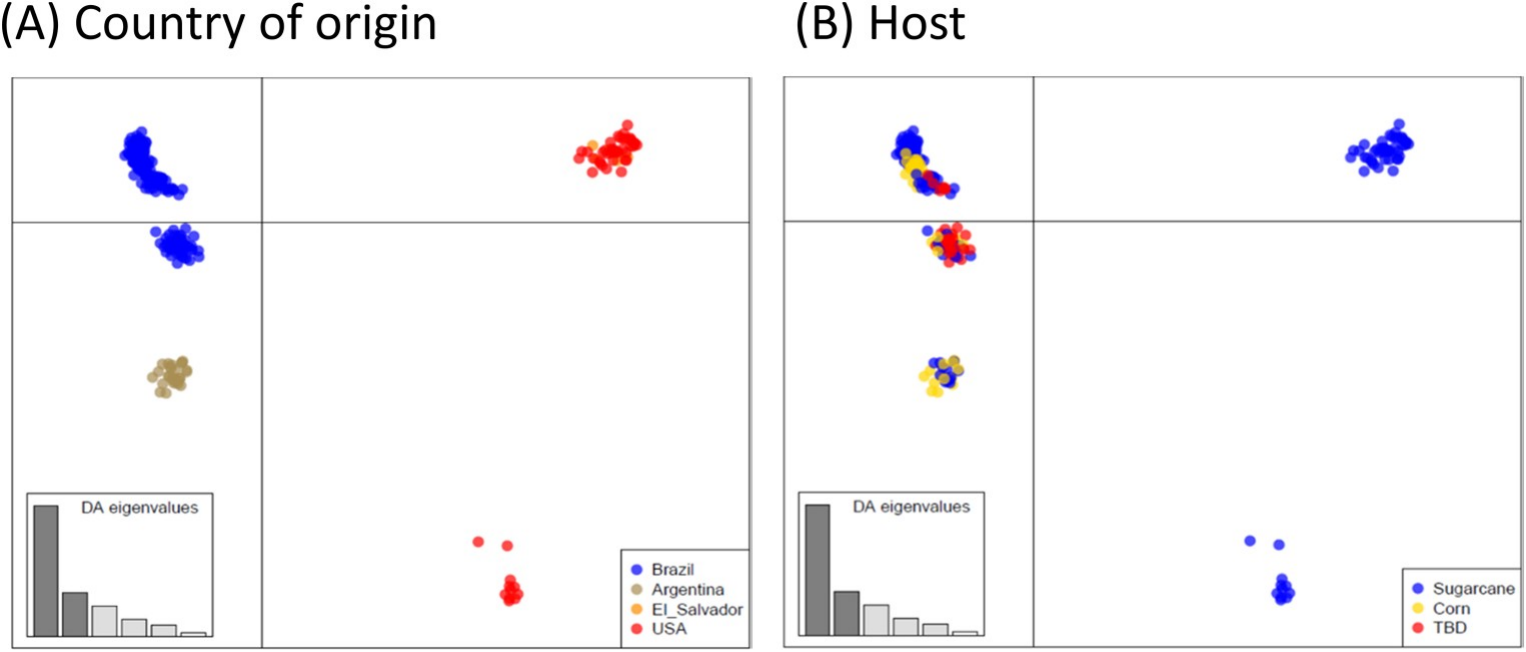
Scatterplots from discriminant analysis of principal components (DAPC) based on 1,331 SNP loci showing the dispersion of 250 *Diatraea saccharalis* individuals across the first two principal components. Individuals (dots) are colored according to (A) their country of origin; and (B) the host crop in which they were collected. Variation represented in x = 44.4% and in y = 15%.

Similarly, the neighbor-joining dendrogram analysis revealed distinct geographically separated groups distinguishing samples from Brazil, Argentina, the U.S. and El Salvador (Fig 2). Interestingly, moths from the north and central-west of Brazil were the most related to the Argentina samples indicating similar founding sources or a connecting route for gene flow. Showing a slightly more significant number of groups, the discriminant analysis of principal components (DAPC) also separates *D*. *saccharalis* samples into four clusters according to the country of origin (Fig 3). The analysis of molecular variance (AMOVAs) supports the structure by geographical regions in which most of the genetic variation was assigned to differences among countries (φst = 0.525, Table 2). The individuals from Argentina and Brazil were more related to each other compared to other locations, confirming that the founding of the two populations is, at least in part, due to related events (i.e., steppingstone or range expansion from similar source populations) (Fig 2). On the other hand, individuals from Florida were more distant from other samples collected in the United States (i.e., Louisiana and Texas), which could be attributed to different sources of the ancestral founding population (Figs 2 and 3). Our finding supports the hypothesis of the colonization of North America by multiple events from different sources, which agrees with Joyce et al. (2014) [26].

**Table 2:** Analyses of Molecular Variance (AMOVA) of *Diatraea saccharalis* populations based on the 1,331 SNP markers. The analysis considered different host-plant species and geographic regions. φ statistics are indices of the amount of differentiation among populations, similar to Wright’s F statistics. d.f. = degrees of freedom.

Source of Variation	D.f	S.S.	M.S.	Sigma	% of variation	φ
Among countries	3	39716.87	13238.96	329.12	52.59	φst = 0.525**
Within countries	192	56972.76	296.73	296.73	47.41	
Total	195	96689.62	495.84	625.86		
Between hosts	1	5052.01	5052.01	66.05	12.27	φst = 0.122**
Within hosts	194	91637.61	472.36	472.36	87.73	
Total	195	96689.63	495.84	538.41		
Among countries	3	39716.87	13238.96	290.58	46.51	φst = 0.549**
Between host within countries	2	3518.63	1759.32	52.79	8.45	φsc = 0.157**
Within hosts	190	53454.13	281.34	281.34	45.04	φct = 0.465*
Total	195	96689.62	495.84	624.71		

Host plants such as maize and sugarcane seem to be a less critical factor structuring *D*. *saccharalis* populations (Fig 3, Table 2). This result agrees with the work of Joyce et al. (2016) [23], which did not find significant genetic divergence among populations of *D*. *saccharalis* collected on maize and sugarcane in El Salvador. Our data indicate that *D*. *saccharalis* populations are undifferentiated in exploiting different hosts, suggesting some level of phenotypic plasticity and can also explain the high number of markers under balancing selection [54].

### Divergence time estimation

Tree topology generated using the ML approach based on *D*. *saccharalis* COI sequences was strongly supported by nuclear markers (S2 Fig). The ML tree divided the data into three distinct groups according to geographic regions. The first split within *D*. *saccharalis* separated Florida from all other populations. A second split separated populations from El Salvador, Mexico, Texas, and Louisiana from those in South America (Brazil and Argentina) indicating a second event of differentiation (S2 Fig). The topology generated from the divergence time analysis based on Bayesian approach revealed a similar pattern of population differentiation. The three different divergence rates used to calibrate the molecular clock rendered different time estimations; however, because there is no specific COI clock rate or fossils records available for *D*. *saccharalis*, all three values were presented (Table 3, S3 Fig). All estimated divergence times place the separation of the three major groups in the period between 427 thousand and 923 thousand years ago. This period precedes the agriculture expansion period in the Americas, which means that the lineages were formed before maize domestication. However, the period is also posterior to great geological transformation in the regions and might be related to climatic oscillation during the Pleistocene [55–57]. The most recent haplotypes were formed within the period of maize domestication and expansion in the Americas.

**Table 3:** Divergence time based on three arthropod mitochondrial divergence rates. Trees are presented in supplemental material S3 Fig.

	2.5%/my	3.54%/my	5.25%/my
Group 1 (Florida/All other populations)	0.923	0.613	0.427
Group 2 (US, El Salvador, Mex/ Brazil, Argentina)	0.685	0.455	0.316

### Hypotheses for the early history of *D*. *saccharalis* colonization

#### Early dispersal strategy—Floating on the Amazon basin rivers

The likely center of origin of *D*. *saccharalis* is the delta region of the Orinoco River, Venezuela, extending to the lower Amazon River [19,58]. Both basins, Orinoco and Amazon, cover an area of approximately 8,380,000 square kilometers (Fig 2, Amazon Basin), an area already large enough to be considered a remarkable dispersal achievement for insects with limited capacity to disperse. The first reports of the movement of *D*. *saccharalis* on infested aquatic grasses floating in the Amazon river are from eyewitness accounts [58]. The movement along the Amazon basin rivers would allow an incremental range expansion out of Venezuela throughout northern Brazil. However, this passive dispersal strategy using the rivers was probably more critical before the association of *D*. *saccharalis* with maize plants which occurred much later for this species.

The early dispersal and the considerable low rate of gene flow possibly created favorable conditions for population isolation and lineage formation as early as ~400–600 thousand years ago. The isolation might have been further enforced by climate oscillation during the late Pleistocene period and prior to the population expansion to more distant parts of the continent. However, the divergence time may vary depending on the clock rate used but consistently points to a period later than any significant geological event (i.e., isthmus of Panama formation ~2.5–3.5 Mya) in the region [59]. An interesting observationis the presence of two distinct lineages of *D*. *saccharalis* in the U.S., which opens questions about two possible colonization events.

#### Pre-Columbian era—The hypothesis for human-aided dispersal by terrestrial routes associated with maize

The dispersal history of *D*. *saccharalis* may have been influenced by human-mediated movements of maize (Fig 2, terrestrial). The ‘*D*. *saccharalis*-maize association’ hypothesis is based on the early occupation of humans in America followed by the establishment of Pre-Columbian civilizations [60,61]. Approximately 14,000 years ago, or possibly earlier, humans began to domesticate plants and animals in the Americas [62–64]. These new cultivation systems associated with the exchange of goods between early American societies may have been responsible for maize dispersal from Mesoamerica to North and South America and the Caribbean following different routes [65–67]. *D*. *saccharalis* may have similarly dispersed to areas beyond its center of origin following maize dispersal in Pre-Columbian America. In North America, a lack of haplotype substructure within the second northern genetic group that includes Texas, Louisiana, Mexico, and El Salvador is a reliable indicator of a great movement by land, which mixed haplotypes in all locations. Populations from Louisiana and Texas were in the same cluster, share most haplotypes, and were strictly related to populations from Central America (El Salvador) indicating a shared route of colonization and gene flow.

Archaeological evidence demonstrates that maize dispersal occurred through the South American lowlands and the Andean region, suggesting two major routes for *D*. *saccharalis* dispersal [65–70]. Analyzing South American lowlands populations (i.e., Brazilian populations), the closer relationships among the populations from the states of Mato Grosso do Sul, Mato Grosso and Tocantins may be associated with early human migrations(Fig 2). This route of human migration was most likely highly dependent on transportation along rivers, probably following the Xingu, Araguaia or/and the Tocantins rivers. These rivers are connected to the Amazon and Orinoco Basins. Alternatively, those haplotypes could have expanded inland from the coast (i.e., São Paulo) coming from the Caribbean. The hypothesis of maritime expansion will be discussed in the next section.

Regarding the *D*. *saccharalis* Argentinian populations, a pattern of sub-structure was found as well. While samples from the Pampa region (i.e., Buenos Aires) and the Dry Pampas (i.e., Tucumán and San Luis) grouped together and showed resemblance to the Brazilian populations, populations from remote parts of the northwest Chaco Serrano (i.e., Jujuy) were genetically differentiated. Together, this suggests that populations from the Pampas and the Dry Pampas might be associated with *D*. *saccharalis* dispersal routes through lowlands, while Jujuy might have a more ancient origin associated with maize dispersal through the Andean region. However, future studies are necessary to confirm this hypothesis.

#### Post-Columbian era—The hypothesis for the human-aided dispersal by maritime routes in association with sugarcane

In recent years, the long-distance movement of goods including sugarcane by maritime routes were of great importance and may explain some of the genetic structure found within the region (Fig 2, maritime routes). One hypothesis to explain the pattern of genetic divergence found in the Florida population could be the commercial movement of plants during the Columbian Exchange period in the 15^th^ and 16^th^ centuries. The Caribbean region was a crossing point at the beginning of European colonization of America, and served to exchange people and food between the Old and the New World [68]. This new commercial center was a favorable environment for the introduction of new diseases and pests that affected the native human population and their native (maize) and introduced (sugarcane) crops. We speculate that the exchange of infested plant material from the center of origin in Venezuela to the Caribbean islands promoted the spread of *D*. *saccharalis* by sea routes, northwards to the United States of America, specifically Florida, as well as southwards to Brazil, specifically to the São Paulo state region. The support for this hypothesis comes from the strong genetic structure within the U.S. that could indicate that the Florida population was colonized by an ancestral population probably from the Caribbean region. Alternatively, gradual range expansion of *D*. *saccharalis* through the Caribbean region could have reached Florida, where the founding population remained relatively isolated throughout the years. More studies including samples from the Caribbean region would be necessary to corroborate this hypothesis.

#### Recent routes of expansion—Agricultural development and routes of colonization of the Brazilian Cerrado

In Brazil, two genetic clusters were derived from the nuclear markers (Fig 3), which could indicate different introduction routes or a gradual process of colonization. The first and largest genetic cluster included the states of São Paulo and Paraná (southeast and south regions), places on the Brazilian coast that could be linked to the first sugarcane plants that arrived in São Paulo state from Caribbean routes. Moreover, when we consider the mitochondrial marker, haplotypes found in São Paulo (Hap3 and Hap7) and Paraná (Hap3, Hap5, and Hap7) were strongly shared with other locations and have a more recent genealogy. Mitochondrial information (COI analysis) supports the hypothesis of a recent process of expansion originating from east to west within Brazil. Also part of this cluster, populations from Goiás and Minas Gerais states (southeast/center-west) were likely colonized by insects present in infested material from São Paulo. It is very likely that the demographic event that allowed the colonization of southeast areas belongs to the same putative route for the colonization of areas further south through the lowlands to Argentina (i.e., the F_ST_ between São Paulo and Buenos Aires was 0.02).

The transportation of plants across states seems to be the most critical pathway for *D*. *saccharalis* dispersion in Brazil countryside, which has been a recent development. This relatively recent introduction of *D*. *saccharalis* in the Brazilian Cerrado, followed by its population expansion may explain the lower allelic richness observed in populations from central Brazil compared to populations collected in the states of São Paulo, where the gene pool was more diverse. Due to its high commercial value during the last decade, sugarcane had a considerable agricultural expansion in areas of the Cerrado. Interesting, however, is that the oldest mitochondrial haplotype was present only in MT (Hap8) and was not shared with any other locations indicating a second route of colonization, and is probably associated with the earlier lowland colonization from the Amazon region. In summary, the combination of haplotypes present in the Cerrado is likely the result of the sugarcane expansion from the east (i.e., São Paulo) and perhaps an earlier pre-sugarcane expansion associated with the movement of maize along the rivers by native populations. More efforts should be made to characterize *D*. *saccharalis* populations from northern parts of Brazil to test this hypothesis.

## Conclusions

*Diatraea saccharalis* is a successful invasive species that colonized habitats with very distinct environmental conditions across the Americas. Likely, multiple introductions of populations over a long time period explain the complex process of colonization of new areas. After an initial period of differentiation that separates different lineages, human migration and trade was likely a major factor in explaining the spread of *D*. *saccharalis* populations throughout the Americas. *Diatraea saccharalis* used maize as the early vehicle for dispersal whereas sugarcane was used in a second historical moment. More information about the genetic background of the Caribbean and South America populations, mainly from Venezuela and Argentina, is needed to support our hypotheses and investigate the lowland and Andean routes of dispersal.

## Supporting information

S1 Fig Loci distribution of *Diatraea saccharalis* populations using the F_ST_ approach implemented in Lositan. Loci plotted in the red area are candidates for being under positive selection while loci plotted in the yellow area are candidates for being under balancing selection.
(TIF)

S2 Fig Phylogeny tree. Maximum likelihood phylogenetic tree was constructed with MEGA X using *D*. *saccharalis* COI sequences. Bootstrap replication values of 1,000 were used, and the number at each node represents the percentage of bootstrap support for each cluster. Data present three distinct clusters separated by geographical locations.
(TIF)

S3 Fig Time tree of *D*. *saccharalis* cytochrome oxidase subunit I (COI) sequences using 5.23%/My divergence rate. (TIFF)

S1 Table Populations of *Diatraea saccharalis* collected and their geographic locations. (DOCX)

S2 Table Haplotype identification of *D*. *saccharalis* collected in BOLD database based on 533 bp fragment of cytochrome oxidase subunit I (COI). (DOCX)

S3 Table Results from the molecular clock test using maximum likelihood method under Hasegawa-Kishino-Yano (1985) (+G). The null hypothesis of equal evolutionary rate throughout the tree was rejected at a 5% significant level (*p* = 0.000). The analysis involved 16 haplotype sequences.
(DOCX)

S4 Table Pairwise values of F_ST_ (below diagonal) and P-values (above) of *Diatraea saccharalis* populations. Bold indicates non-significant P-values (P > 0.05).
(DOCX)
