# Supplementary figures and images for: Diatraea saccharalis history of colonization in the Americas. The case for human-mediated dispersal
Source: PLoS One. 2019 Jul 24;14(7):e0220031. doi: 10.1371/journal.pone.0220031 (PMC6656350; doi:10.1371/journal.pone.0220031)

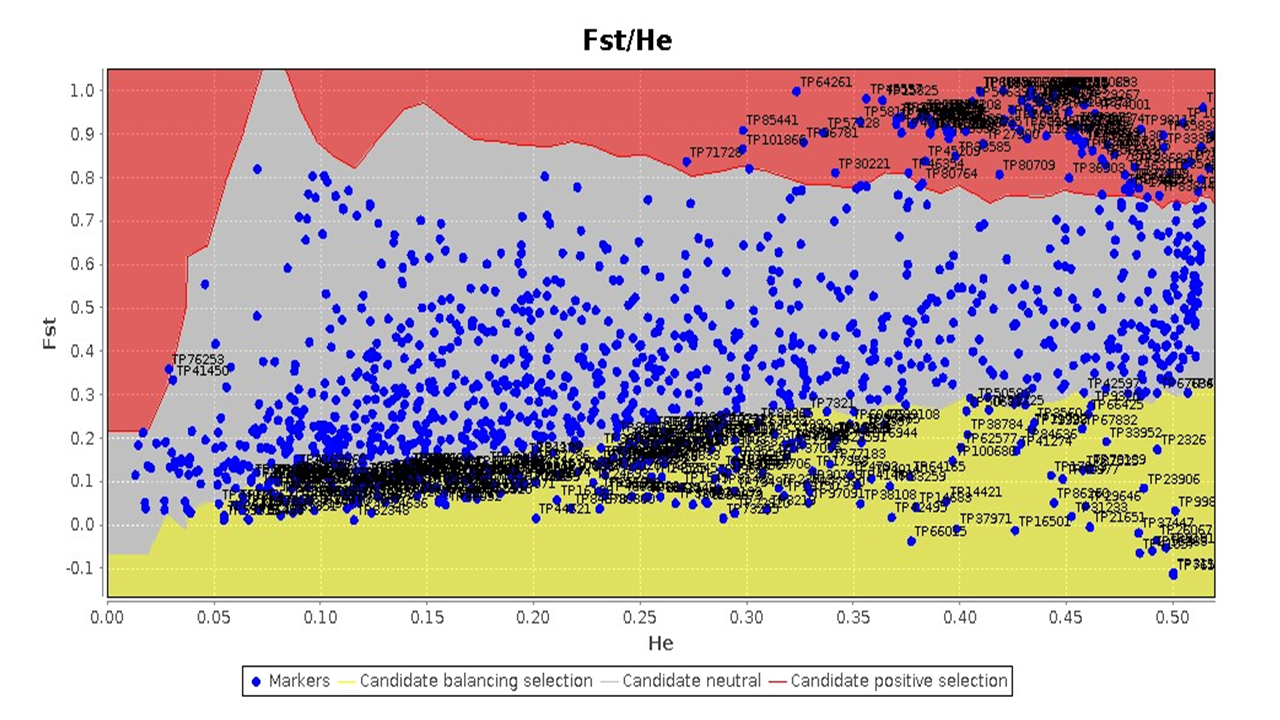

Supplement: S1 Fig — Loci plotted in the red area are candidates for being under positive selection while loci plotted in the yellow area are candidates for being under balancing selection. (TIF) [file pone.0220031.s001.tif]

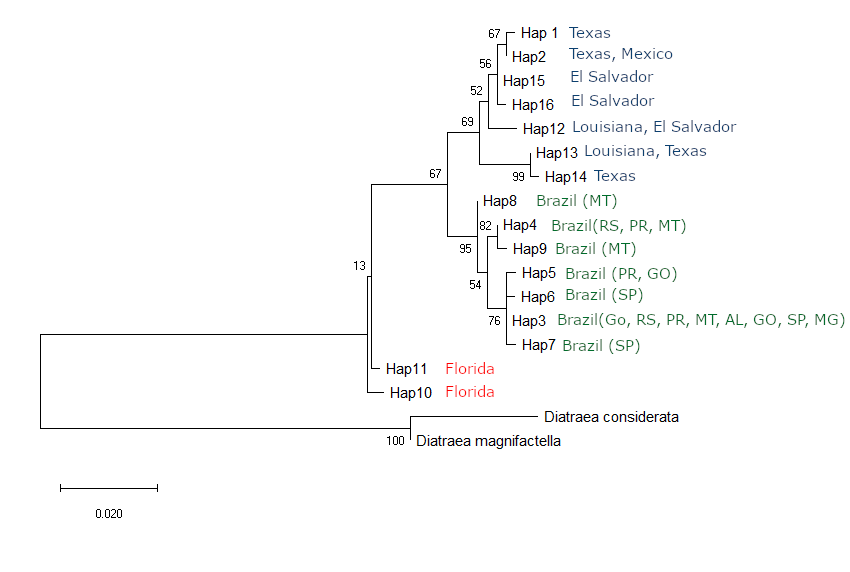

Supplement: S2 Fig — Bootstrap replication values of 1,000 were used, and the number at each node represents the percentage of bootstrap support for each cluster. Data present three distinct clusters separated by geographical locations. (TIF) [file pone.0220031.s002.tif]

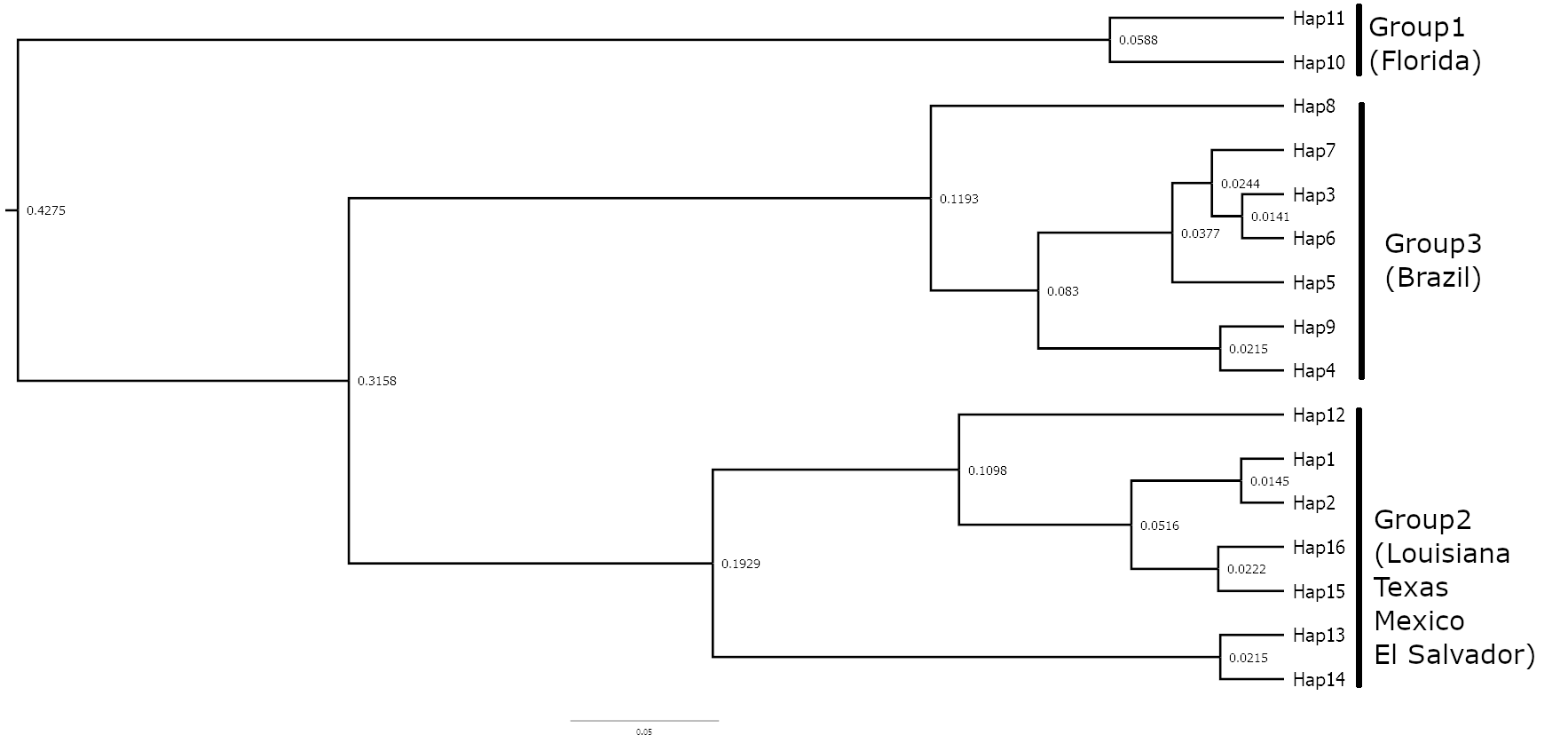

Supplement: S3 Fig — (TIFF) [file pone.0220031.s003.tiff]
