# Supplementary material for: Diatraea saccharalis history of colonization in the Americas. The case for human-mediated dispersal
Source: PLoS One. 2019 Jul 24;14(7):e0220031. doi: 10.1371/journal.pone.0220031 (PMC6656350; doi:10.1371/journal.pone.0220031)
Supplement: S1 Table — (DOCX) [file pone.0220031.s004.docx]

S1 Table. Populations of *Diatraea saccharalis* collected and their geographic locations.

| Code | Population (Country) | Population (State) | Population (City) | Host | Latitude | Longitude | Number of  Individuals |
| --- | --- | --- | --- | --- | --- | --- | --- |
| LaCocha_Co | Argentina | Tucumán | La Cocha | Maize | -27.7680453 | -65.5841519 | 10 |
| LaCruz_Su | Argentina | Tucumán | La Cruz | Sugarcane | -29.1783205 | -56.6378331 | 5 |
| Jujuy_Su | Argentina | Jujuy | Jujuy | Sugarcane | -24.1843397 | -65.302177 | 7 |
| Perga_Co | Argentina | Buenos Aires | Pergamino | Maize | -33.8912831 | -60.5745999 | 2 |
| Qui_Co | Argentina | San Luis | Quines | Maize | -32.2337053 | -65.8055325 | 4 |
| Adam_Su | Brazil | São Paulo | Adamantina | Sugarcane | -21.717868 | -51.0152294 | 1 |
| Araras_Su | Brazil | São Paulo | Araras | Sugarcane | -22.3604911 | -47.3798391 | 2 |
| Goias_TBD | Brazil | Goiás | Goiás | TBD | -16.6868824 | -49.2647885 | 4 |
| Inac_Co | Brazil | Goiás | Inaciolândia | Maize | -18.4873555 | -49.9892163 | 1 |
| Jabo_Su | Brazil | São Paulo | Jaboticabal | Sugarcane | -21.2525138 | -48.3256762 | 52 |
| Minas_TBD | Brazil | Minas Gerais | Minas Gerais | TBD | -19.9465885 | -43.9698479 | 1 |
| Morr_Co | Brazil | Goiás | Morrinhos | Maize | -17.734945 | -49.1208516 | 4 |
| Morr_Su | Brazil | Goiás | Morrinhos | Sugarcane | -17.734945 | -49.1208516 | 1 |
| MS_TBD | Brazil | Mato Grosso do Sul | Mato Grosso do Sul | TBD | -17.7342695 | -49.1193721 | 9 |
| MT_TBD | Brazil | Mato Grosso | Mato Grosso | TBD | -15.5889647 | -56.0814921 | 5 |
| PAf_Su | Brazil | Tocantins | Pedro Afonso | Sugarcane | -8.9707508 | -48.1733686 | 3 |
| Parana_TBD | Brazil | Paraná | Paraná | TBD | -25.4244287 | -49.2653819 | 3 |
| Pira_Co | Brazil | São Paulo | Piracicaba | Maize | -22.7342864 | -47.6480644 | 25 |
| Pira_Su | Brazil | São Paulo | Piracicaba | Sugarcane | -22.7342864 | -47.6480644 | 22 |
| Rib_Su | Brazil | São Paulo | Ribeirão Preto | Sugarcane | -21.1704008 | -47.8103238 | 4 |
| Rondo_Co | Brazil | Mato Grosso | Rondonópolis | Maize | -16.4654757 | -54.6387229 | 3 |
| SHG_Su | Brazil | Goiás | Santa Helena de Goiás | Sugarcane | -17.8119748 | -50.5981252 | 4 |
| SP_TBD | Brazil | São Paulo | São Paulo | TBD | -23.5505199 | -46.6333094 | 18 |
| Uber_Co | Brazil | Minas Gerais | Uberlândia | Maize | -18.8936275 | -48.221351 | 1 |
| ElNilo_Su | El Salvador | El Nilo | El Nilo | Sugarcane | -13.3951138 | -88.8809583 | 4 |
| ElPais_Su | El Salvador | El Paisnal | El Paisnal | Sugarcane | 13.9755122 | -89.217002 | 2 |
| FLA_Su | USA | Florida | unknown | Sugarcane | 26.6845104 | -80.6675577 | 5 |
| BGlade_Su | USA | Florida | Belle Glade | Sugarcane | 26.6845104 | -80.6675577 | 9 |
| LA_Su | USA | Louisiana | unknown | Sugarcane | NA | NA | 4 |
| Louis_Su | USA | Louisiana | Louisiana | Sugarcane | 30.9842977 | -91.9623327 | 15 |
| Beaum_Su | USA | Texas | Beaumont | Sugarcane | 30.080174 | -94.1265562 | 13 |
| Wesl_Su | USA | Texas | Weslaco | Sugarcane | 26.1595194 | -97.9908366 | 7 |
| TBD = To be determined (information not available). | | | |  |  |  |  |
