# Supplementary material for: Diatraea saccharalis history of colonization in the Americas. The case for human-mediated dispersal
Source: PLoS One. 2019 Jul 24;14(7):e0220031. doi: 10.1371/journal.pone.0220031 (PMC6656350; doi:10.1371/journal.pone.0220031)
Supplement: S2 Table — (DOCX) [file pone.0220031.s005.docx]

**S2 Table. Haplotype identification of *D. saccharalis* collected in BOLD database based on 533 bp fragment of cytochrome oxidase subunit I (COI).**

| Hap 1 | BBLOC1560-11  BBLOD166-11 | U.S.(Texas) |
| --- | --- | --- |
| Hap 2 | BBLOC1565-11, GBGL12822-13, GBGL12823-13  GBGL12824-13, GBGL12825-13, GBGL12826-13  GBGL12827-13, GBGL12828-13 | U.S.(Texas), Mexico, |
| Hap 3 | GBGL14922-14, GBGL14923-14, GBGL14924-14, GBGL14925-14, GBGL14926-14,GBGL14929-14, GBGL14930-14, GBGL14931-14, GBGL14937-14, GBGL14938-14, GBGL14939-14, GBGL14940-14, GBGL14941-14, GBGL14942-14, GBGL14943-14, GBGL14944-14, GBGL14945-14, GBGL14946-14, GBGL14947-14, GBGL14948-14, GBGL14949-14, GBGL14950-14, GBGL14951-14, GBGL14952-14, GBGL14953-14, GBGL14954-14, GBGL14955-14, GBGL14956-14, GBGL14957-14, GBGL14958-14, GBGL14959-14, GBGL14960-14, GBGL14961-14, GBGL14962-14, GBGL14963-14, GBGL14965-14, GBGL14966-14, GBGL14967-14, GBGL14968-14, GBGL14969-14, GBGL14970-14, GBGL14971-14, GBGL14972-14, GBGL14973-14, GBGL14974-14, GBGL14975-14, GBGL14976-14, GBGL14977-14, GBGL14978-14, GBGL14980-14, GBGL14981-14, GBGL14982-14, GBGL14983-14, GBGL14984-14, GBGL14985-14, GBGL14986-14, GBGL14987-14, GBGL14988-14, GBGL14989-14, GBGL14990-14, GBGL14991-14, GBGL14992-14, GBGL14993-14, GBGL14994-14, GBGL14995-14, GBGL14996-14, GBGL14998-14, GBGL14999-14, GBGL15000-14, GBGL15002-14, GBGL15003-14, GBGL15004-14, GBGL15005-14, GBGL15006-14, GBGL15007-14, GBGL15008-14, GBGL15009-14, GBGL15010-14, GBGL15011-14, GBGL15012-14, GBMIN30577-13, GBMIN30578-13, GBMIN30579-13, GBMIN30580-13, GBMIN30581-13, GBMIN30582-13,GBMIN30583-13, GBMIN30584-13, GBMIN30585-13, GBMIN30586-13, GBMIN30589-13, GBMIN30590-13, GBMIN30591-13, GBMIN30603-13, GBMIN30604-13, GBMIN30605-13, GBMIN30606-13, GBMIN30607-13, GBMIN30608-13, GBMIN30609-13, GBMIN30610-13, GBMIN30611-13, GBMIN30612-13, GBMIN30613-13, GBMIN30615-13, GBMIN30616-13, GBMIN30617-13 | Brazil (GO, RS, PR, MT, AL, GO, SP, MG) |
| Hap 4 | GBGL14927-14, GBGL14928-14, GBGL14932-14, GBGL14933-14, GBGL14934-14, GBGL14935-14, GBGL14979-14, GBGL15013-14, GBGL15018-14, GBGL15019-14, GBGL15020-14, GBGL15021-14, GBMIN30587-13, GBMIN30588-13, GBMIN30614-13 | Brazil (RS, PR, MT) |
| Hap5 | GBGL14936-14, GBGL14964-14 | Brazil (PR, GO) |
| Hap 6 | GBGL14997-14 | Brazil (SP) |
| Hap 7 | GBGL15001-14 | Brazil (SP) |
| Hap 8 | GBGL15014-14 | Brazil (MT) |
| Hap 9 | GBGL15015-14 | Brazil (MT) |
| Hap 10 | GBGL17309-15 | U.S. (Florida) |
| Hap 11 | GBGL17310-15 | U.S. (Florida) |
| Hap 12 | GBGL17312-15 | Louisiana, El Salvador |
| Hap 13 | GBGL17313-15 | Louisiana, Texas |
| Hap 14 | GBGL17316-15 | Texas |
| Hap 15 | GBMIN79384-17 | El Salvador |
| Hap 16 | GBMIN79396-17 | El Salvador |
| Outgroup 1 | KP259615.1_Diatraea |  |
| Outgroup 2 | GBMTG1702-16 |  |
