# Supplementary material for: Diatraea saccharalis history of colonization in the Americas. The case for human-mediated dispersal
Source: PLoS One. 2019 Jul 24;14(7):e0220031. doi: 10.1371/journal.pone.0220031 (PMC6656350; doi:10.1371/journal.pone.0220031)
Supplement: S3 Table — The null hypothesis of equal evolutionary rate throughout the tree was rejected at a 5% significant level (p = 0.000). The analysis involved 16 haplotype sequences. (DOCX) [file pone.0220031.s006.docx]

**S3 Table. Results from the molecular clock test using Maximum Likelihood method under Hasegawa-Kishino-Yano (1985) (+G).** The null hypothesis of equal evolutionary rate throughout the tree was rejected at a 5% significant level (*p*= 0.000). The analysis involved 16 haplotype sequences.

|  | **lnL** | **Parameters** | **(+G)** |
| --- | --- | --- | --- |
| With clock | -35138.305 | 20 | 0.050 |
| Without clock | -938.924 | 34 | 0.05 |
