# Supplementary material for: Diatraea saccharalis history of colonization in the Americas. The case for human-mediated dispersal
Source: PLoS One. 2019 Jul 24;14(7):e0220031. doi: 10.1371/journal.pone.0220031 (PMC6656350; doi:10.1371/journal.pone.0220031)
Supplement: S4 Table — Bold indicates non-significant P-values (P > 0.05). (DOCX) [file pone.0220031.s007.docx]

**S4 Table. Pairwise values of F_ST_ (below diagonal) and P-values (above) of *Diatraea saccharalis* populations. Bold indicates non-significant P-values (P > 0.05).**

|  | Buenos Aires | El Nilo | El Pais | Florida | Goias | Jujuy | Louisiana | M. Gerais | Mato G. Sul | Mato Grosso | Paraná | San Luis | São Paulo | Texas | Tocantins | Tucuman |
| --- | --- | --- | --- | --- | --- | --- | --- | --- | --- | --- | --- | --- | --- | --- | --- | --- |
| Buenos Aires |  |  |  |  |  |  |  |  |  |  |  |  |  |  |  |  |
| El Nilo | 0.381 |  |  |  |  |  |  |  |  |  |  |  |  |  |  |  |
| El Paisnal | 0.216 | 0.014 |  |  |  |  |  |  |  |  |  |  |  |  |  |  |
| Florida | 0.329 | 0.180 | 0.146 |  |  |  |  |  |  |  |  |  |  |  |  |  |
| Goias | 0.050 | 0.204 | 0.177 | 0.242 |  |  |  |  |  |  |  |  |  |  |  |  |
| Jujuy | 0.043 | 0.280 | 0.241 | 0.270 | 0.075 |  |  |  |  |  |  |  |  |  |  |  |
| Louisiana | 0.418 | 0.089 | 0.056 | 0.261 | 0.304 | 0.348 |  |  |  |  |  |  |  |  |  |  |
| Minas Gerais | 0.049 | 0.337 | 0.173 | 0.339 | 0.015 | 0.100 | 0.406 |  |  |  |  |  |  |  |  |  |
| Mato G. do Sul | 0.048 | 0.231 | 0.201 | 0.260 | 0.033 | 0.066 | 0.323 | 0.028 |  |  |  |  |  |  |  |  |
| Mato Grosso | 0.054 | 0.240 | 0.205 | 0.256 | 0.036 | 0.064 | 0.327 | 0.059 | 0.041 |  |  |  |  |  |  |  |
| Paraná | 0.068 | 0.296 | 0.217 | 0.328 | 0.019 | 0.094 | 0.392 | 0.031 | 0.034 | 0.048 |  |  |  |  |  |  |
| San Luis | 0.034 | 0.411 | 0.334 | 0.349 | 0.067 | 0.052 | 0.444 | 0.119 | 0.065 | 0.068 | 0.107 |  |  |  |  |  |
| São Paulo | 0.026 | 0.134 | 0.124 | 0.160 | 0.014 | 0.037 | 0.210 | 0.005 | 0.018 | 0.021 | 0.003 | 0.033 |  |  |  |  |
| Texas | 0.399 | 0.067 | 0.037 | 0.233 | 0.298 | 0.336 | 0.015 | 0.396 | 0.316 | 0.317 | 0.376 | 0.419 | 0.211 |  |  |  |
| Tocantins | 0.060 | 0.297 | 0.234 | 0.310 | 0.025 | 0.075 | 0.384 | 0.038 | 0.030 | 0.029 | 0.039 | 0.101 | 0.012 | 0.367 |  |  |
| Tucuman | 0.039 | 0.272 | 0.237 | 0.268 | 0.114 | 0.044 | 0.350 | 0.129 | 0.100 | 0.095 | 0.129 | 0.047 | 0.063 | 0.337 | 0.106 | 0 |
